# Breastfeeding and circulating immunological markers during the first 3 years of life: the DIABIMMUNE study

**DOI:** 10.1007/s00125-021-05612-2

**Published:** 2021-11-27

**Authors:** Maija E. Miettinen, Jarno Honkanen, Sari Niinistö, Outi Vaarala, Suvi M. Virtanen, Mikael Knip, the DIABIMMUNE Study Group

**Affiliations:** 1 Department of Public Health and Welfare, Health and Well-Being Promotion Unit, Finnish Institute for Health and Welfare, Helsinki, Finland; 2 Research Program for Clinical and Molecular Metabolism, Faculty of Medicine, University of Helsinki, Helsinki, Finland; 3 Faculty of Social Sciences, Unit of Health Sciences, Tampere University, Tampere, Finland; 4 Tampere University Hospital, Research, Development and Innovation Center, Tampere, Finland; 5 Center for Child Health Research, Tampere University and Tampere University Hospital, Tampere, Finland; 6 Children’s Hospital, University of Helsinki and Helsinki University Hospital, Helsinki, Finland; 7 Folkhälsan Research Center, Helsinki, Finland; 8 Research Program for Clinical and Molecular Metabolism, University of Helsinki, Helsinki, Finland; 9 Department of Pediatrics, Tampere University Hospital, Tampere, Finland

**Keywords:** Breastfeeding, Calprotectin, Chemokine, Children, Cytokine, Growth factor, Human β defensin-2, Immunological marker, Type 1 diabetes

## Abstract

**Aims/hypothesis:**

Our aim was to study the association between duration of breastfeeding and circulating immunological markers during the first 3 years of life in children with HLA-conferred susceptibility to type 1 diabetes.

**Methods:**

We performed a longitudinal analysis of 38 circulating immunological markers (cytokines, chemokines and growth factors) in serum samples from Finnish (56 individuals, 147 samples), Estonian (56 individuals 148 samples) and Russian Karelian children (62 individuals, 149 samples) at 3, 6, 12, 18, 24 and 36 months of age. We also analysed gut inflammation markers (calprotectin and human β defensin-2) at 3 (*n* = 96) and 6 months (*n* = 153) of age. Comparisons of immunological marker medians were performed between children who were breastfed for 6 months or longer vs children who were breastfed for less than 6 months.

**Results:**

Breastfeeding for 6 months or longer vs less than 6 months was associated with lower median of serum immunological markers at 6 months (granulocyte-macrophage colony-stimulating factor [GMCSF], macrophage inflammatory protein [MIP-3α]), 12 months (IFN-α2, vascular endothelial growth factor, GMCSF, IFN-γ, IL-21), 18 months (FGF-2, IFN-α2) and 24 months of age (CCL11 [eotaxin], monocyte chemoattractant protein-1, TGFα, soluble CD40 ligand, IL-13, IL-21, IL-5, MIP-1α) (all *p* < 0.01) but not at 36 months of age. Breastfeeding was not associated with gut inflammation markers at 3 and 6 months of age.

**Conclusions/interpretation:**

Children who were breastfed for 6 months or longer had lower medians for 14 immunological markers at one or more age points during the first 2 years of life compared with children who were breastfed for less than 6 months. The clinical meaning of the findings is not clear. However, the present study contributes to the understanding of immunological differences in children that have been breastfed longer, and thus provides a mechanistic suggestion for the previously observed associations between breastfeeding and risk of type 1 diabetes.

**Graphical abstract:**

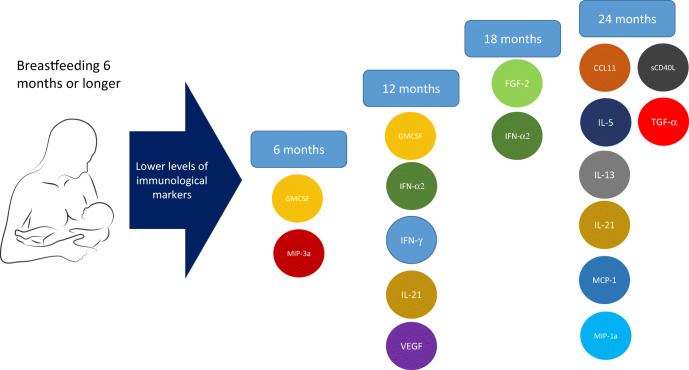

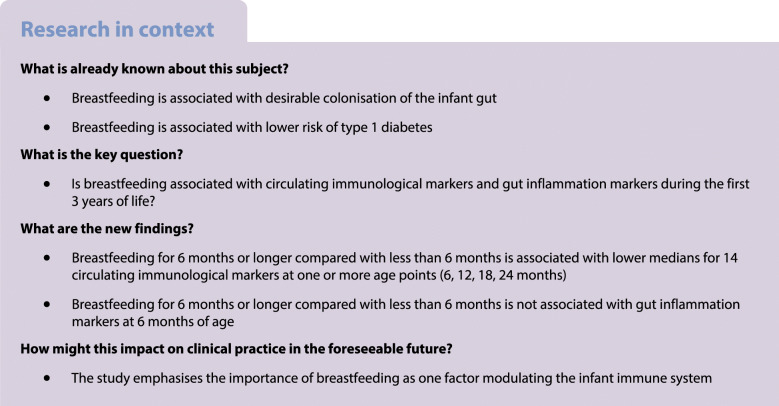



## Introduction

The prevalence of childhood-onset immune-mediated diseases, including type 1 diabetes, asthma and allergy, has considerably increased especially in high- and middle-income countries during the past decades. Epidemiological data suggest that early-life environmental exposures are key determinants of these diseases [1]. The so-called hygiene hypothesis has been suggested to explain the disease increase by diminishing early-life microbial and parasite infections, which may be needed to prevent harmful immune responses later in life, but underlying mechanisms are unclear [2].

Breastfeeding after birth has been reported to support the immature immune system of an infant through various immunomodulating components present in breast milk including anti-inflammatory cytokines. Breastfeeding is strongly associated with the development of gut microbiota, providing a desirable microbial colonisation of the gut, as demonstrated by higher levels of *Bifidobacterium* species in breastfed infants [3]. Additionally, breastfeeding has been associated with lower diversity and slower maturation of the gut microbiome [3].

Breastfeeding associates with lower concentration of serum and gut inflammation markers in infants after birth [4] but these associations have not been consistent or thoroughly investigated. Preterm infants have defective maturation of the immune system including lower production of various cytokines. Cytokines present in breast milk have been implicated in helping infants to form a sufficient immune response [5]. However, it is not known whether longer breastfeeding affects and possibly continues to benefit the developing immune system. Breastfeeding has been associated with lower risk of type 1 diabetes or islet autoimmunity in several studies [6], although the mechanism remains open to debate.

The aim of this study was to evaluate the association between breastfeeding and both circulating immunological markers and gut inflammation markers during the first 3 years of life.

## Methods

### Study population

All new-born infants born between September 2008 and February 2011 in one hospital in Finland, two hospitals in Estonia and two hospitals in Russian Karelia were screened for HLA-conferred susceptibility to type 1 diabetes. Children with genotypes that increase the risk of the disease were invited to the birth cohort of the DIABIMMUNE study and followed prospectively from birth up to 3 years of age. From 835 children originally included in the study, 38 were excluded due to incomplete data, leaving 797 children (386 in Finland, 322 in Estonia, and 89 in Russia) [7]. Of the 797 children, analysis of circulating immunological markers were performed in children that had unthawed serum samples available (56 children and 147 samples from Finland, 56 children and 148 samples from Estonia and 62 children and 149 samples from Russian Karelia) from when children were 3, 6, 12, 18, 24 and 36 months of age. Gut inflammation markers (calprotectin and human β defensin-2) were analysed in the 3 (*n* = 96) and 6 month (*n* = 153) samples. Breastfeeding status was recorded at each time point. The local ethics committees (Ethics committee, Helsinki and Uusimaa Hospital District; Ethics Review Committee on Human Research of the University of Tartu; and Ethics committee, Ministry of Health and Social Development, Karelian Republic of the Russian Federation) approved the study and parents provided written informed consents.

### HLA genotyping

The cord blood samples from the new-born infants were screened for HLA DR/DQ genotypes associated with increased risk for type 1 diabetes. Children positive for *DR3-DQ2* (DQA1*05-DQB1*02) and/*or DR4-DQ8* (DRB1*04:01/2/4/5/8-DQB1*0302/4) without protective haplotypes were eligible for the study. Children carrying any of the following protective haplotypes were excluded: DQB1*03:01, DQB1*06:02, DQB1*06:03, DRB1*04:03, (DR14)-DQB1*05:03 and (DR7)-DQA1*02:01-DQB1*03:03.

### Serum immunological markers

The concentrations of circulating cytokines, chemokines and growth factors were analysed from unthawed serum samples with Luminex technology using the 38-plexed Milliplex MAP Kit (cat. no. HCYTMAG-60K-PX38) according to the manufacturer’s recommendations (Merck-Millipore Corp., Billerica, MA, USA). Analyses were performed with single reactions using undiluted serum samples. Quantification of the markers was performed with the Bio-Plex 200 Luminex instrument and Bio-Plex Manager software (Bio-Rad, Sweden). The concentration of each marker was determined from an eight-point standard curve using five-parameter logistic regression. The minimum detectable concentration (MinDC) was determined for each marker separately using the lowest concentration on the standard curve linear phase (MinDC = C(low) + 2SD). The samples below the MinDC were given a value of 50% of MinDC.

Comparisons of immunological marker medians were performed between children who were breastfed for 6 months or longer vs children who were breastfed for less than 6 months. The numbers of children breastfed for less than 3 months or for 12 months or longer were low, thus preventing meaningful comparisons at the age of 3 or 12 months.

### Statistical analyses

Serum immunological marker and gut inflammation marker data are expressed as medians. Differences in serum and gut inflammation marker medians were compared using the Mann–Whitney *U* test. *p* values <0.01 were considered statistically significant. The analyses were performed using IBM SPSS Statistics for Windows, Version 27.0 (Released 2020; IBM Corp. Armonk, NY, USA).

## Results

The mean duration of exclusive breastfeeding was 1.1 months in Finland, 1.4 months in Estonia and 3.3 months in Russian Karelia (*p* < 0.001). The total mean duration of breastfeeding was 9.1 months in Finland, 9.3 months in Estonia and 7.4 months in Russian Karelia (*p* = 0.046).

Breastfeeding for 6 months or longer compared with less than 6 months was associated with lower median of serum immunological markers at 6 months (granulocyte-macrophage colony-stimulating factor [GMCSF], macrophage inflammatory protein [MIP]-3α), 12 months (IFN-α2, vascular endothelial growth factor [VEGF], GMCSF, IFN-γ, IL-21), 18 months (FGF-2, IFN-α2) and 24 months of age (eotaxin [CCL11], monocyte chemoattractant protein-1 [MCP-1], TGF-α, soluble CD40 ligand [sCD40L], IL-13, IL-21, IL-5, MIP-1α) (all *p* < 0.01) (Table 1). Borderline association (*p* < 0.05) was found between breastfeeding for 6 months or longer with lower median of several serum immunological markers at 6, 12, 18 and 24 months of age. No associations were found at 36 months of age.

**Table 1:** Differences in circulating immunological markers at 6, 12, 18, 24 and 36 months of age in children breastfed for less than 6 months compared with children breastfed for 6 months or longer

Marker	6 months				12 months				18 months				24 months				36 months			
	*N*	Median	IQR	*p* value	*N*	Median	IQR	*p* value	*N*	Median	IQR	*p* value	*N*	Median	IQR	*p* value	*N*	Median	IQR	*p* value
CCL11																				
Breastfeeding <6 months	22	450	212	0.69	29	500	214	0.56	12	466	276	0.73	15	636	394	0.008**	18	733	454	0.28
Breastfeeding ≥6 months	51	443	306		59	458	261		38	466	245		35	467	257		33	580	376	
FGF-2																				
Breastfeeding <6 months	22	86.1	89.3	0.15	29	97.0	50.2	0.043	12	118	80.2	0.009**	15	145	85.8	0.031	18	154	105	0.18
Breastfeeding ≥6 months	51	60.8	82.7		59	72.3	61.8		38	66.6	54.4		35	73.7	92.4		33	116	83.3	
IFNα-2																				
Breastfeeding <6 months	22	17.8	40.4	0.027	29	36.6	35.4	0.005**	12	53.2	33.0	0.006**	15	48.9	66.0	0.012	18	48.0	80.7	0.34
Breastfeeding ≥6 months	51	4.44	24.1		59	25.1	37.4		38	20.1	36.3		35	19.9	29.8		33	32.0	57.8	
MCP-1																				
Breastfeeding <6 months	22	688	329	0.51	29	616	369	0.79	12	614	124	0.60	15	700	147	0.006**	18	660	220	0.16
Breastfeeding ≥6 months	51	754	301		59	662	326		38	584	217		35	497	239		33	553	305	
TGF-α																				
Breastfeeding 6 months	22	6.10	11.5	0.016	27	8.64	10.4	0.013	12	9.76	20.2	0.025	15	10.8	9.90	0.005**	17	6.80	9.34	0.57
Breastfeeding ≥6 months	51	3.52	2.93		56	4.76	8.85		38	5.04	4.62		35	5.44	5.88		33	6.09	7.98	
VEGF																				
Breastfeeding <6 months	10	610	219	0.90	18	513	45.4	0.003**	8	511	279	0.73	6	678	1150	0.60	4	730	784	0.64
Breastfeeding ≥6 months	24	639	582		36	500	18.2		21	526	498		17	727	455		9	596	971	
sCD40L																				
Breastfeeding <6 months	22	4100	1730	0.71	29	4860	2420	0.38	12	4490	2460	0.53	15	5390	4160	0.008**	18	5710	4040	0.25
Breastfeeding ≥6 months	51	3930	1780		59	3810	2520		38	3560	2010		35	3730	3350		33	4150	6250	
GMCSF																				
Breastfeeding <6 months	22	33.0	36.4	0.001**	28	76.7	55.6	<0.001**	12	63.5	48.3	0.67	15	113	67.1	0.023	18	90.2	93.5	0.15
Breastfeeding ≥6 months	48	13.8	20.0		59	37.4	19.5		38	52.4	71.7		35	62.0	73.9		33	84.6	79.4	
IFN-γ																				
Breastfeeding <6 months	22	7.70	6.51	0.034	28	16.0	13.1	<0.001**	12	22.7	22.3	0.059	15	16.3	24.2	0.045	18	16.8	9.27	0.63
Breastfeeding ≥6 months	50	4.68	4.50		58	11.7	9.10		38	11.1	9.77		35	12.1	11.9		33	16.7	8.81	
MIP-3α																				
Breastfeeding <6 months	22	66.0	21.6	0.002**	29	69.0	15.9	0.027	12	70.0	37.2	0.30	15	65.8	29.9	0.29	18	67.7	20.2	0.44
Breastfeeding ≥6 months	51	52.4	15.1		59	61.5	17.6		38	67.7	17.4		35	68.2	21.4		33	63.2	21.2	
IL-13																				
Breastfeeding <6 months	21	5.89	6.02	0.31	29	13.0	9.82	0.067	12	13.1	21.5	0.040	15	15.5	15.1	0.008**	18	12.5	8.78	0.096
Breastfeeding ≥6 months	45	5.06	4.43		59	9.82	6.28		37	8.13	5.87		35	9.01	8.49		33	9.02	8.62	
IL-21																				
Breastfeeding <6 months	22	32.9	58.4	0.017	29	53.0	58.1	0.001**	12	51.8	43.7	0.052	15	43.7	44.1	0.004**	18	28.5	33.0	0.096
Breastfeeding ≥6 months	50	5.62	18.9		59	23.7	30.6		38	30.7	29.4		35	24.1	27.1		33	26.3	23.8	
IL-5																				
Breastfeeding <6 months	21	1.21	1.08	0.33	28	2.57	2.01	0.48	12	2.39	2.71	0.019	15	2.81	2.68	0.006**	18	2.17	1.25	0.28
Breastfeeding ≥6 months	48	0.99	0.81		58	2.39	1.40		37	1.87	0.97		35	1.84	1.36		33	1.98	1.30	
MIP-1α																				
Breastfeeding <6 months	22	20.3	11.3	0.11	28	25.7	16.3	0.17	12	26.9	8.31	0.022	15	27.8	9.57	0.002**	18	25.7	6.76	0.54
Breastfeeding ≥6 months	50	17.5	4.96		58	23.9	6.39		38	21.1	6.96		35	23.5	7.74		33	25.7	3.22	

**p < 0.01 (Mann–Whitney *U* test)

Altogether, 78 and 116 children had both breastfeeding status and gut inflammation marker results available at 3 months of age and 6 months of age, respectively. Breastfeeding for 3 or 6 months or longer compared with less than 3 or 6 months was not associated with gut inflammation markers (human β defensin-2 and calprotectin) at 3 or 6 months of age.

Altogether, nine children seroconverted to islet autoimmunity and one child developed type 1 diabetes. Given the low number of children with islet autoimmunity or type 1 diabetes and given the high individual variation of inflammation marker concentrations, meaningful analyses according to disease outcomes could unfortunately not be performed.

## Discussion

We found associations between circulating immunological markers and breastfeeding at several time points during the first 24 months of life. These results provide novel information on the relationship between breastfeeding and the immune system during early childhood.

The strengths of the study include repeated measurements of a large array of circulating cytokines, chemokines and growth factors during the first years of life. Given the fact that the DIABIMMUNE study comprises children from Finland, Estonia and Russia, it would be interesting to determine whether the association between circulating immunological markers and breastfeeding would show differences within these three countries. The sample size was, however, so small in each individual country that meaningful analyses could not be performed. It was seen, however, that the duration of exclusive breastfeeding was higher in Russian Karelia than in Finland and Estonia, although the total duration of breastfeeding showed no large differences between the three countries.

IFN-α has been reported to be associated with several autoimmune diseases including type 1 diabetes. Increased expression of genes stimulated by INF-α have been seen in pancreatic biopsies taken from individuals with recent-onset type 1 diabetes compared with islets from control organ donors [8]. Both the Finnish DIPP [9] and the German BABYDIET study [10] reported that the IFN-α signature is temporally increased prior to the development of autoantibodies. In the present study, difference in IFN-α2 was seen at 12 and at 18 months of age, and also a nominal difference at 6 and at 24 months of age, strongly suggesting that breastfeeding modulates IFN-α2 production. More detailed analyses are needed, however, to understand the potential clinical significance of this association.

Previous studies have reported higher calprotectin concentrations in breastfed children compared with formula-fed children [11]. We did not find any difference in gut inflammation markers (human β defensin-2 and calprotectin) when comparing children that were breastfed for 3 or 6 months or longer with children that were breastfed less than 3 or 6 months. It would have been interesting to analyse whether there would be differences at other age points. Unfortunately, however, data for gut inflammation marker concentrations were available only at 3 and at 6 months of age.

The possibility of finding at least some of the differences just by chance cannot be ruled out. However, whenever a statistical difference was observed in the current study, the median of the immunological marker was consistently lower in children that were breastfed for 6 months or longer compared with children that were breastfed for less than 6 months. Possible generalisability of the results to a non-risk population can, unfortunately, not be sorted out in this study, since the DIABIMMUNE study inclusion criteria included only children carrying increased genetic risk for type 1 diabetes.

Breastfeeding for 6 months or longer was associated consistently with lower medians of altogether 14 serum immunological markers at one or more time points during the first 2 years of life. At 36 months of age, no differences were seen in serum immunological markers in relation to earlier breastfeeding history. The clinical meaning of the findings is not clear, because no direct association with clinical type 1 diabetes could be determined in this study setting and because previous studies have not defined normal levels of serum immunological markers during infancy. However, the present study contributes to the understanding of immunological differences in children who have been breastfed for a longer period, and accordingly provides a potential mechanism to the association previously observed between breastfeeding and risk of type 1 diabetes.

## Data Availability

The authors confirm that, for approved reasons, some access restrictions apply to the datasets generated during and/or analysed during the current study underlying the findings. Researchers interested in using the data are required to follow the terms of a number of clauses designed to ensure the protection of privacy and compliance with relevant regulations. Data are available upon request due to ethical restrictions, pending approval from the relevant ethical committees.

## Abbreviations

CCL11: Eotaxin
GMCSF: Granulocyte-macrophage colony-stimulating factor
MCP-1: Monocyte chemoattractant protein-1
MinDC: Minimum detectable concentration
MIP: Macrophage inflammatory protein
sCD40L: Soluble CD40 ligand
VEGF: Vascular endothelial growth factor
